# Synthesis, Structure, and Magnetic Properties of Pseudo-One-Dimensional μ3-oxo Manganese (III) Phosphate Exhibiting Magnetization Steps at Low Temperatures

**DOI:** 10.1063/4.0000870

**Published:** 2025-10-27

**Authors:** Kulugammana Gedera Sanjaya Ranmohotti

**Affiliations:** Governors State University, University Park, IL, USA

## Abstract

It has been fascinating to investigate solids containing magnetic nanostructures of different size and geometry. Transition metal (TM) oxy compounds are of great interest for their structural versatility. During our exploratory synthesis, targeting new compounds with confined magnetic nanostructures, a new manganese (III) phosphate was isolated. This pseudo-one-dimensional m3-oxo Ba2Mn3O2(PO4)3 crystalline compound is an intriguing example of the mixed-framework solids featuring, in particular, a periodic array of well-defined TM-oxide nanostructures that are embedded in oxyanion matrix. It crystallizes in an orthorhombic space group (Pnma, No. 62) with the m3-oxo magnetic chains packed in a semi- hexagonal array. Dark brown crystals were grown by the dissolution and nucleation processes from the mixture of the corresponding oxides (Mn2O3, P4O10, BaO) in a molten-salt media, which is made of eutectic RbCl/NaCl flux (mp = 540 oC). The crystal structure was resolved by the single crystal X-ray diffraction methods, and it shows an extended lattice characterized by the [Mn3P3O14]4- slabs with barium cations residing in the interslab space. The manganese oxide polyhedra share both edge- and vertex oxygen atoms to form an infinite chain propagating along the c axis. The chain is conceptually “chelated” by phosphate anions through sharing its non-bridging oxygen atoms and coincides with the longest dimension of the column crystal. Each chain consists of the [Mn O ] ] 17- unit made of two MnO square pyramids and one MnO octahedron. These three manganese oxide polyhedral units share a common oxygen atom, O(1) and O(2). In the presence of μ3 oxygen atoms, fascinating wave like interconnected triangula^3^r la^1^tt^3^ice i^¥^s formed and assume to be behavin^5^g like magnetically frustrating system. The s^6^ubject of this fascin_3_atin_1_g_3_in_¥_vestigation is around the [Mn O ] 17- chain, its structural separation and, in principle, electronic insulation by phosphate anions, PO 3-. The magnetic susceptibility data shows Curie-Weiss paramagnetic behavior in the range of ca. 10- 300K and a possible antiferro-to-ferro4magnetic transition at ∼10 K. This compound shows hysteresis in magnetization versus DC field scans, and the hysteresis loops show steps at regular intervals of magnetic field, the diagnostic evidence of field-tuned quantum tunneling of magnetization. AC magnetic susceptibility studies on this compound have been performed in the 2.0-8.0 K range in a 3.0 Oe field oscillating at frequencies up to 1500 Hz. The field- dependent magnetic studies reveal that step-like magnetic hysteresis loops at low temperatures. In this presentation, we will discuss the results of synthesis, structure, magnetic property measurements, as well as structure and property correlation studies of this m3-oxo Manganese (III) Phosphate.

